# Assessing the Impact of Community Engagement Interventions on Health Worker Motivation and Experiences with Clients in Primary Health Facilities in Ghana: A Randomized Cluster Trial

**DOI:** 10.1371/journal.pone.0158541

**Published:** 2016-07-20

**Authors:** Robert Kaba Alhassan, Edward Nketiah-Amponsah, Nicole Spieker, Daniel Kojo Arhinful, Tobias F. Rinke de Wit

**Affiliations:** 1 Amsterdam Institute for Global Health and Development, University of Amsterdam, Amsterdam, Netherlands; 2 Department of Epidemiology, Noguchi Memorial Institute for Medical Research, University of Ghana, Legon, Accra, Ghana; 3 Department of Economics, University of Ghana, Legon, Accra, Ghana; 4 PharmAccess Foundation, Amsterdam, Netherlands; Vanderbilt University, UNITED STATES

## Abstract

**Background:**

Health worker density per 1000 population in Ghana is one of the lowest in the world estimated to be 2.3, below the global average of 9.3. Low health worker motivation induced by poor working conditions partly explain this challenge. Albeit the wage bill for public sector health workers is about 90% of domestic government expenditure on health in countries such as Ghana, staff motivation and performance output remain a challenge, suggesting the need to complement financial incentives with non-financial incentives through a community-based approach. In this study, a systematic community engagement (SCE) intervention was implemented to engage community groups in healthcare quality assessment to promote mutual collaboration between clients and healthcare providers, and enhance health worker motivation levels. SCE involves structured use of existing community groups and associations to assess healthcare quality in health facilities. Identified quality gaps are discussed with healthcare providers, improvements made and rewards given to best performing facilities for closing quality care gaps.

**Purpose:**

To evaluate the effect of SCE interventions on health worker motivation and experiences with clients.

**Methods:**

The study is a cluster randomized trial involving health workers in private (n = 38) and public (n = 26) primary healthcare facilities in two administrative regions in Ghana. Out of 324 clinical and non-clinical staff randomly interviewed at baseline, 234 (72%) were successfully followed at end-line and interviewed on workplace motivation factors and personal experiences with clients. Propensity score matching and difference-in-difference estimations were used to estimate treatment effect of the interventions on staff motivation.

**Results:**

Intrinsic (non-financial) work incentives including cordiality with clients and perceived career prospects appeared to be prime sources of motivation for health staff interviewed in intervention health facilities while financial incentives were ranked lowest. Intervention health facilities that were assessed by female community groups (Coef. = 0.2720, p = 0.0118) and informal groups with organized leadership structures like Artisans (Coef. = 0.2268, p = 0.0368) associated positively with higher intrinsic motivation levels of staff.

**Conclusion:**

Community-based approach to health worker motivation is a potential complementary strategy that needs policy deliberation to explore its prospects. Albeit financial incentives remain critical sources of staff motivation, innovative non-financial approaches like SCE should complement the latter.

## Background

Health sector human resource remains a critical input factor in health service delivery. A 2013 progress report by the World Health Organization (WHO) titled “*A universal truth*: *no health without a health workforce*” intimated the important role played by health workers in the attainment of universal health coverage and health sector goals. Though the global health workforce situation is relatively better in most developed countries, statistics on poor countries remain appalling particularly in Africa [1].

According to the WHO estimates, a minimum density threshold of 22.8 skilled health professionals per 10,000 people is required globally to provide the most basic health coverage but 83 countries (mostly in Africa) fall below this threshold [1]. Even though over 59 million health workers are recorded globally, the distribution of this workforce within and between countries is disproportionate with the health needs and disease burdens of these countries. African countries bear the brunt of health workforce shortage requiring approximately 139% increase to meet the growing population health needs [2].

In Ghana, the health worker density per 1000 population is estimated to be 2.32 [3], below the global average of 9.3 [2]. Low staff motivation levels induced by prevailing poor working conditions is a major contributory factor for the disparities in health workforce distribution in Ghana [4–7].

Determinants of health worker motivation have been explored in the literature from varying perspectives. Bennette and Franco [8] defined health worker motivation as an individual’s degree of willingness to exert and maintain an effort towards organizational goals. The role of societal and community context in health worker motivation has been demonstrated in the argument by Bennette and Franco [8] that health worker motivation entails an internal psychological process that is influenced by the organizational and larger societal context. Theories on worker motivation are broadly categorized into four namely: need-based, cognitive process, behavioral and job based theories. Motivation theories that explain employees’ quest to satisfy their needs through work were categorized as need-based theories in Jex and Britt [9]. These theories contend that workers attain motivation by being able to attain their needs, ranging from basic physiological needs to higher level ones such as self-actualization.

Cognitive process theories of worker motivation argue that motivation at the workplace, including healthcare facilities, is a function of cognitive process of evaluation where employees strive to achieve a perceived balance between their efforts at the workplace and rewards given or anticipated [9]. Behavioral theories suggest that motivation can be promoted when beneficial employee’s behaviour is rewarded and non-beneficial behaviour is discouraged through prudent punishment [9]. Job-based theories of motivation, however, maintain that the design of an employee’s job in itself can determine motivation levels, thus a job can be motivating by its design and content [9].

Even though these theories of motivation have limitations [9], they remain relevant to this study and contemporary discussions on employee motivation within the health sector [8]. Moreover, previous related studies on Ghana [4–7,10,11] and elsewhere [12–15] have established the relevance of these theories in health worker motivation.

Empirical conclusions on the determinants of health worker motivation differ. Mathauer and Imhoff [12] indicated that besides the demand for equity in employee effort: reward ratios and financial incentives, intrinsic/non-financial motivation factors such as opportunity for promotion, ability to satisfy professional conscience and available work logistics are significant determinants of worker motivation. For the purposes of this paper, the researchers utilized Bennette and Franco’s [8] conceptual framework on intrinsic and extrinsic motivational factors.

As part of efforts towards enhancing staff motivation levels in Ghana, work incentives such as salary increment and payment of extra duty hours allowance are often implemented [3,10,12]. Albeit these incentives are relevant, mainly relying on them without adequate complementary intrinsic (non-financial) incentives has proved insufficient in sustaining staff motivation and performance output [4,11,12,13].

Moreover, limited health budgets of most countries in Africa including Ghana pose a significant challenge for these countries to rely solely on financial rewards to motivate health staff which do not guarantee desired performance output [7]. Although over 90% of domestic expenditure on health is on the wage bill (staff salaries) in countries such as Ghana [16], staff productivity and quality of healthcare delivery remain a major challenge in health facilities [4,11]. Since health staff motivation is determined by factors beyond material and financial rewards, there is the need to explore the possible benefits of intrinsic (non-financial) motivational packages which could be potentially cost effective and sustainable in the long term.

Studies have shown that besides financial incentives, good working relationships between staff and clients promotes staff motivation and quality of healthcare delivery, especially at the primary healthcare level [17,18]. Health workers who perceive a sense of duty to the community they serve are more likely to be intrinsically motivated and have better working experience with clients [19,20].

This paper hypothesizes that Systematic Community Engagement (SCE) in health is a potential approach that could help enhance relationships between service providers and communities and promote health worker intrinsic motivation level.

Community engagement as defined by Morgan and Lifshay [21], cited in Alhassan et al [22], is dynamic relationships and dialogue between community members and local health professionals with varying degrees of community and higher level health authorities’ involvement in decision-making and control.

Though the concept of community engagement in health is not entirely new in Ghana [23–28], empirical evidence of its relevance and relationship with health worker motivation is quite limited and gray. Available information on community engagement in health is largely reported in annual reports, project reports and media briefs usually without exploring the associations with health worker motivation. Besides this study, there is no known randomized cluster trial in Ghana on this topic.

Moreover, in Ghana community engagement in health is often limited to the Community-based Health Planning and Services (CHPS) concept which by design does not deliberately engage existing community groups to assess health service quality using the SCE approach.

The SCE interventions, evaluated in this study, entail a structured step-by-step and cyclical process of engaging community groups/associations in assessing health service quality in their nearest health facility.

Intervention health facilities where the SCE interventions were implemented were all accredited by the National Health Insurance Authority (NHIA). The NHIA is the regulatory authority under the Ministry of Health (MoH) responsible for credentialing of health facilities willing to render services to subscribers of Ghana’s National Health Insurance Scheme (NHIS). Thirty-two (32) healthcare facilities were randomly assigned to treatment and control facilities. Out of the 32 treatment facilities, 16 from each of the two study regions were assigned to receive SCE interventions which lasted for nearly one year, costing approximately US$ 280.00 per a round of SCE. Detailed description of the SCE implementation process has been published by the authors in Alhassan et al [22,23].

This paper evaluates impact of the SCE interventions on staff motivation levels and experiences with clients in the intervention health facilities. The hypothesis is that health facilities that are assessed by community groups will have better motivated staff and enhanced experiences with clients than control facilities.

## Methods

### Study design

This study is a randomized cluster trial involving clinical and non-clinic health workers in private (*n* = 38) and public (*n* = 26) primary health facilities; *n* represents the respective sample sizes. Primary health facilities are operationally defined in this study to mean clinics and health centres, according to the Ghana Health Service (GHS) categorizations. Health workers with at least 6 months’ working experience were eligible to participate in the study. Randomization into intervention and control groups was done at the health facilities level, not at the staff level.

### Study population and setting

The study was conducted in the Greater Accra and Western regions of Ghana in 16 administrative districts. Greater Accra region is predominantly urban with a population of about 4 million people and hosts approximately 20.6% of the estimated 53,000 health workforce in Ghana [3]. Out of the nearly 4,000 accredited health facilities in Ghana, 416 are in Greater Accra region while 438 are in Western region [29]. Western region is predominantly rural with a population of over 2 million served by 7.7% of the total health workforce in Ghana [3].

### Sampling

A total of 333 questionnaires were randomly administered to eligible health staff in 64 sampled health facilities during baseline survey conducted between March and May, 2012 including mop ups. Out of this number, 324 questionnaires were correctly filled and returned representing 97% return rate. Out of the 324 staff interviewed at baseline, 234 (72%) were successfully followed between August and October, 2014 including mop ups. The 90 drop-out staff (28%) could not be followed because of transfers, deaths, resignations and retirements. One health facility was lost to follow-up due to closure thus reducing the follow-up sample size to 63 clinics.

### Data collection

Structured questionnaires were used to collect information on factors that motivate or constrain health workers to deliver quality healthcare services to clients. Background information of staff and their experiences with clients during health service delivery were also explored. Staff were asked to rank their motivation levels on 19 workplace motivation proxies using a four-point Likert scale from 1 = “very disappointing” to 4 = “very satisfactory”.

The study was piloted in one private and one public health facilities in the Greater Accra region to enhance the scientific rigor and value of the full-scale study. The pilot helped determine the feasibility and acceptability of the study methodology and data collection instruments prior to full-scale implementation. The pilot facilities were all excluded from the actual baseline and follow-up surveys.

A total of 16 data collectors and field supervisors were recruited and trained for three days out of which 10 data collectors and 2 field supervisors were selected for the surveys. Five data collectors and one supervisor each were assigned to the Greater Accra and Western regions to interview sampled health workers using structured questionnaire of 205 closed and open ended questions; 70 of these questions are directly related to this current paper. Average duration per interview was 55 minutes.

### Ethical considerations

Ethical clearance was obtained from the Ghana Health Service (GHS) Ethical Review Committee (ERC) (clearance number: GHS-ERC: 18/5/11). Written informed consent was obtained from health facility heads, the district and regional health directorates, and individual respondents. Coding was done after data cleaning to anonymize the staff’s responses.

### Statistical analysis

Analysis was done on “intention to treat” basis. Only data from staff interviewed at baseline and followed-up was used for the final analysis [30]. The STATA statistical software version 12.0 (StataCorp, College Station. Texas USA) was used for all analysis. Pearson Chi-square (*X*^2^) and Fisher’s exact tests were performed, as appropriate, to ascertain differences in socio-demographic characteristics of staff in intervention and control facilities. The t-test was used to compare parameters on staff experiences with clients in intervention and control facilities.

Iterated Principal Factor (IPF) analysis (with orthogonal varimax rotation, Kaiser off) was performed to group the 19 workplace motivational markers into 5 factors. The 5 factor-analyzed parameters were predicted and named as follows: (i) physical work environment and resource availability; (ii) financial and extrinsic incentives; (iii) intrinsic incentives and cordiality with fellow staff and patients; (iv) career prospects and opportunity for further education; (v) staff strength and workload.

The average reliability coefficient for the 19 Likert scale items was tested and found to be above the 0.70 rule of thumb [31]. The four point Likert scales were intuitively determined and the five factor-analyzed factors were predicted and named based on the conceptual framework by Bennette and Franco [8] on determinants of workplace motivation. Difference-in-difference (DiD) test was performed to ascertain the mean rating differences by staff in intervention and control facilities using the pooled baseline and follow-up datasets [32]. Means and standard errors of the five motivation factors were bootstrapped and estimated by linear regression.

Even though the overall study from which this paper emanates from is a randomized cluster trial by design, the health staff sampled to ascertain their perceptions of workplace motivation factors were not randomly assigned to the treatment and control arms of the study. Randomization into control and intervention groups was done at the health facility level, not the staff level. Hence, staff who by chance were found in intervention or control clinics were interviewed at random (i.e. cluster randomization).

Propensity score matching (psmatch2) was employed to determine the treatment effect of the SCE interventions on staff motivation markers without introducing selection bias [33,34]. Potential effect of covariates such as staff gender, age, education, professional category, monthly salary, marital status, facility ownership (private or public) and religious affiliation were corrected.

## Results

### Background information of staff and work conditions

Out of the 234 staff successfully followed-up, 56% were in control facilities and 44% in intervention facilities; in terms of regional distribution, 128 (55%) were from Greater Accra region and 106 (45%) from Western region. In terms of ownership, 53% were from private facilities and 47% from public facilities. Females dominated males in both intervention (males = 40%; females = 60%) and control facilities (males = 30%; females = 70%). The mean age of respondents at baseline was 37(SD = 14) and 38(SD = 12) at follow-up. Average age of staff in intervention facilities was 38.3(SD = 14.4) and 36.5 (SD = 13.4) in control facilities.

The proportion of staff performing clinical roles (clinical staff) in 2012 reduced from 85.5 to 65.4 in 2014; these staff were perhaps reassigned to non-clinical duties during the follow-up survey which explains the corresponding increase in the proportion of staff performing non-clinical roles from 14.5 in 2012 to 34.6 in 2014 (p = 0.000).

Between baseline and follow-up, the average monthly salaries of health staff increased significantly; the proportion of staff who received monthly salary >GHC1, 300 (approx. US$ 370.0) increased from 4.7 in 2012 to 12.8 in 2014 (p = 0.002). Likewise, the proportion of staff who received additional work allowance from their health facilities increased from 16.5 in 2012 to 25.7 in 2014 (p = 0.017).

The percentage of staff that belong to a professional association increased from 60.1 in 2012 to 74.4 in 2014 (p = 0.007). No significant differences were found in the educational, marital and religious characteristics of staff between baseline and follow-up. Moreover, staff travel time to work depending on means of transport did not change significantly between 2012 and 2014 (see Table 1).

**Table 1:** Profile of health staff and work conditions at baseline and follow-up.

	**Baseline (n = 234)**		**Follow-up (n = 234)**		**Diff.**	**p-value**
**Staff characteristics/work conditions**	**Obs**	**Proportion(95% CI)**	**Obs**	**Proportion(95% CI)**		
Staff with at least tertiary education	234	74.8 (69.2 80.4)	234	77.4 (71.9 82.8)	2.6	0.516
Clinical staff^++^	234	85.5 (80.9 90.0)	234	65.4 (59.2 71.5)	-20.1	0.000**
Non-clinical staff^+++^	234	14.5 (10.0 19.1)	234	34.6 (28.5 40.8)	20.1	0.000**
Married staff	233	43.8 (37.4 50.2)	232	45.3 (38.8 51.7)	1.5	0.748
Christian religion	233	96.6 (94.2 98.9)	233	95.7 (93.1 98.3)	-0.9	0.631
Monthly salary >GHC 1,300	232	4.7 (2.0 7.5)	226	12.8(8.4 17.2)	8.1	0.002^‡^
Receive additional work allowance	224	16.5 (11.6 21.4)	230	25.7(20.0 31.3)	9.2	0.017^‡^
Engaged in part time work besides regular work	227	9.3 (5.5 13.0)	230	7.8(4.3 11.3)	-1.5	0.586
Belong to a professional association	183	60.1 (52.9 67.3)	183	74.4(67.1 81.8)	14.3	0.007**
Report late to work at most once in a week	115	78.3 (70.6 85.9)	106	70.8(62.0 79.6)	-7.5	0.469
Average travel time to work in minutes if:	**Obs**	^a^**Mean(95% CI)**	**Obs**	**Mean(95% CI)**	**Diff.**	**p-value**
Walking	108	10.5 (8.6 12.3)	112	11.9 (9.4 14.5)	1.4	0.3591
Bicycle	2	4.0 (-8.7 16.7)	4	17.5 (2.3 32.7)	13.5	0.1343
Motorcycle	4	14.0 (-7.2 35.2)	5	10.3 (-9.6 47.6)	-3.7	0.7132
Public transport	84	39.2 (33.0 45.4)	81	43.6 (36.4 50.8)	4.4	0.3565
Personal car	31	31.0 (20.6 41.4)	25	36.2 (35.1 47.4)	5.2	0.4850
Extra work hours by staff in a day (in minutes)	117	58.1 (14.8 120.0)	230	69.6 (47.8 91.3)	11.5	0.6027
Staff age in years	227	37.3 (35.5 39.1)	234	37.7 (36.1 39.3)	0.4	0.7314

**Source:** WOTRO-COHEiSION Ghana Project (2014).

^‡^Fisher’s exact test (p<0.05).

**Pearson Chi-square test (p<0.05).

^a^Mean testing done with the independent t-test at 95% confidence level.

^+^Observations are the pooled responses of staff at baseline and follow-up.

^++^Staff who performed clinical roles.

^+++^staff who performed non-clinical roles.

### Staff personal experiences with clients

**Fig 1:**
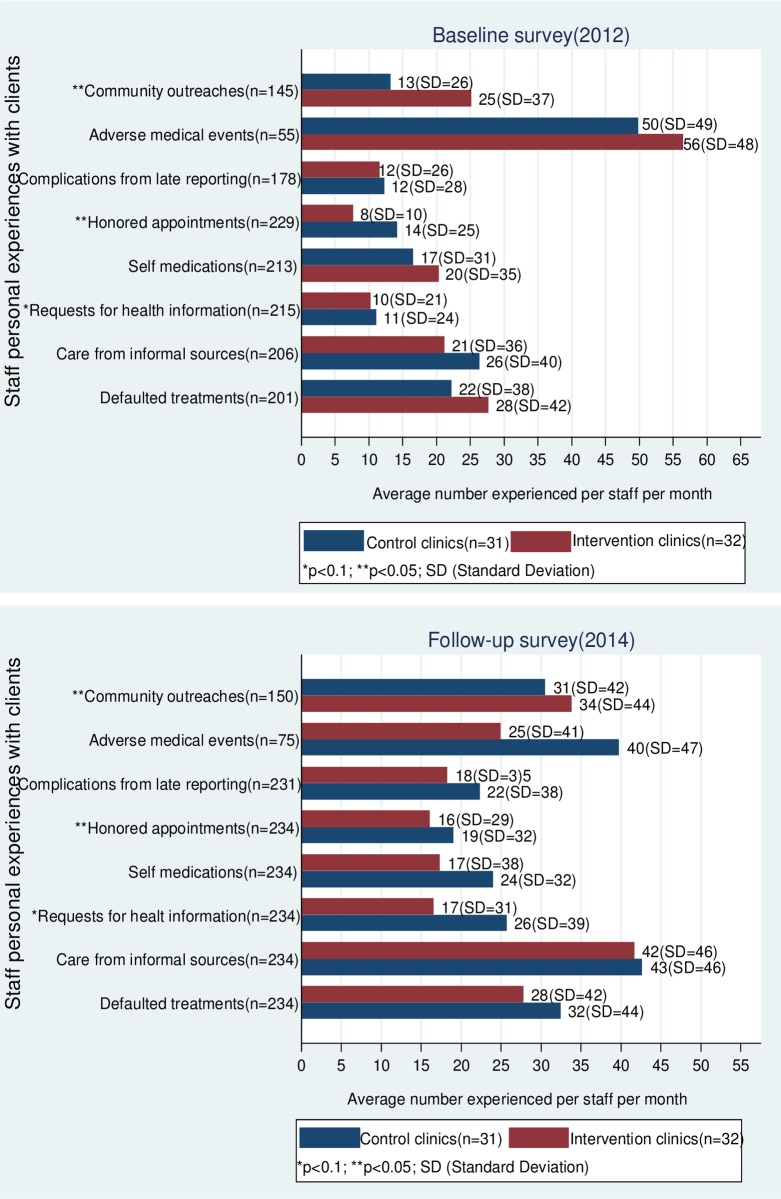
Staff personal experiences with clients in control and intervention facilities. **Source:** WOTRO-COHEiSION Ghana Project (2014).

The results show that in intervention facilities, the average number of community outreaches conducted by staff (mostly community health workers) in a month increased from 25 (SD = 37) at baseline to 34 (SD = 44) during follow-up (p = 0.0105). Staff in control facilities reported relatively lower number of community outreaches per month (see Fig 1).

The average number of clients who honored their medical appointment dates in a month improved in 2014; staff in intervention facilities experienced an average of 8 (SD = 10) honored appointments in a month in 2012 compared to 16 (SD = 29) in 2014; although staff in control facilities also reported increased number of honored appointments by clients, the figures were relatively lower (from 14 in 2012 to 19 in 2014).

These improvements in staff perceptions in intervention facilities might not necessarily be ascribed to the effect of the SCE interventions alone since institutional level developments such as increased outpatient and inpatient services and health infrastructure might have informed staff experiences.

Responses from health staff seemed to suggest a general trend of increased client curiosity on their health conditions. During baseline, staff in intervention facilities experienced an average of 10 (SD = 21) clients asking health workers questions concerning their health conditions in a month compared to 17 (SD = 31) clients at follow-up (p = 0.0486); staff in control facilities recorded relatively better staff experiences in this regard (from 11 in 2012 to 26 in 2014).

Number of staff who experienced at least an adverse medical event in a month increased from 55 in 2012 to 75 in 2014. In intervention health facilities, the average number of adverse medical events experienced per staff per month decreased from 56 (SD = 48) in 2012 to 25 (SD = 47) in 2014. Even though staff in control facilities also experienced a reduction in number of adverse events per month, the improvement was lower (from 50(SD = 49) in 2012 to 40(SD = 47) in 2014). Besides the possible effect of the SCE interventions on these reports by staff, the time lag (two years) between baseline and follow-up might have informed significant quality improvements and patient safety protocols that influenced staff experiences.

As shown in Fig 1, it appears more clients in 2014 sought care from informal caregivers (e.g. spiritualists, traditional healers) before visiting the health facility; in a month, staff in intervention facilities experienced an average of 21 (SD = 36) clients engaged in this practice in 2012 compared to 42 (SD = 46) clients in 2014. Staff in control facilities experienced an average of 26 (SD = 40) clients seeking care from informal sources in a month in 2012 compared to 43 (SD = 46) in 2014.

The average number of clients defaulting in medical treatment protocols increased in control facilities from an average of 22 (SD = 38) clients in a month in 2012 to 32 (SD = 44) in 2014. Staff in intervention facilities did not experience significant increases in number of defaulting clients. Likewise, the average number of clients reporting with history of self-medication reduced in intervention facilities from 20 (SD = 35) in a month in 2012 to 17 (SD = 38) in 2014. Control facilities increased from 17 (SD = 31) in a month in 2012 to 24 (SD = 32) in 2014, but the differences were not statistically significant (see Fig 1).

### Effect of SCE interventions on staff motivation

**Fig 2:**
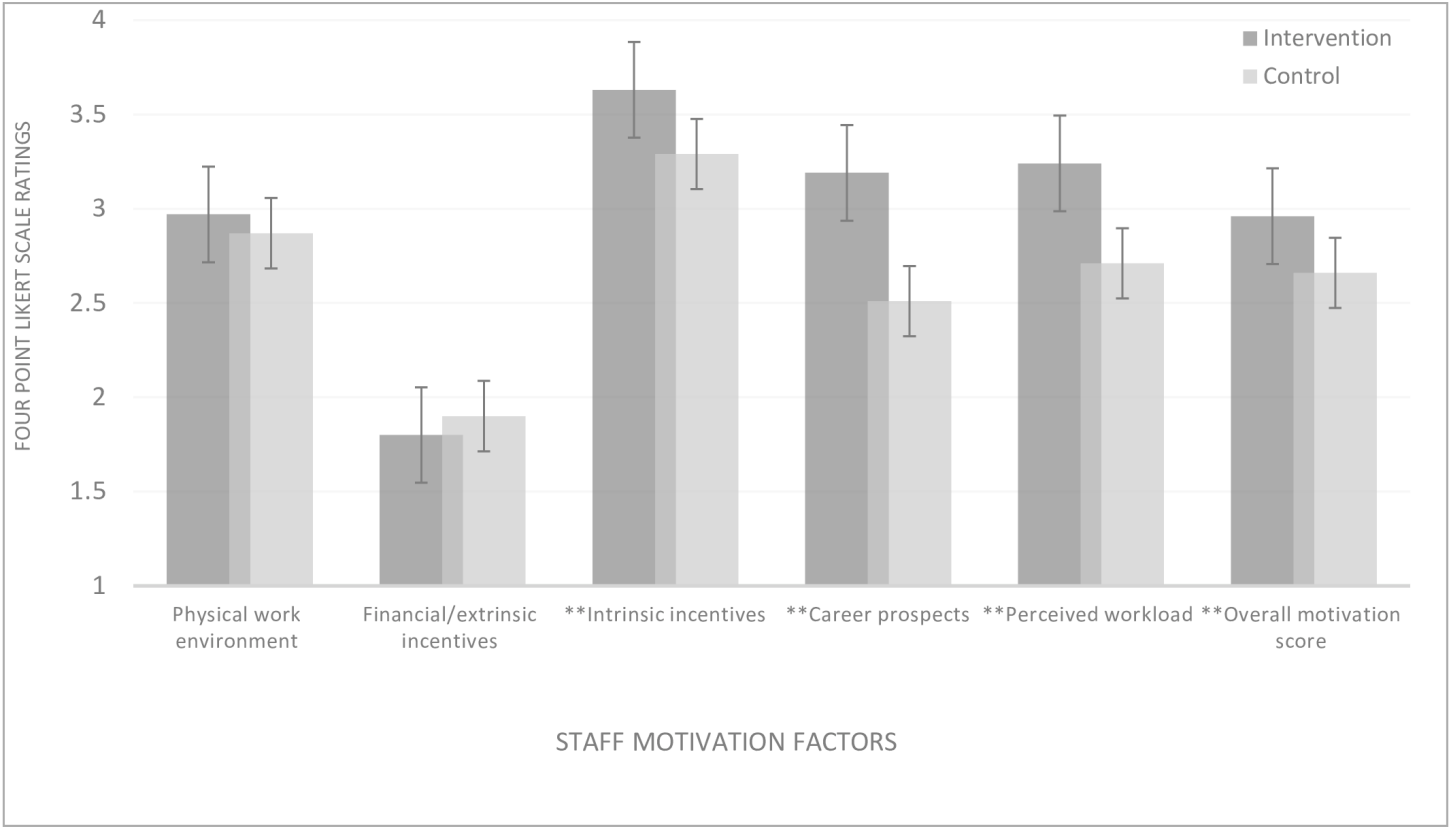
Workplace motivating factors in treatment and control facilities. **Source:** WOTRO-COHEiSION Ghana Project (2014); **p<0.0001 (two tailed test of hypothesis using t-test). **Note1:** Mean testing was based on pooled baseline and follow-up responses in 2012 and 2014 respectively. Means were derived from a four-point Likert scale from 1 = “Very disappointing” to 4 = “Very satisfactory”. High summated scores per staff motivation area depict better satisfaction with work conditions and *vice-versa*. **Note2:** Extrinsic motivation is derived from financial and material work conditions of a job (e.g. salary increment, promotion, accommodation etc.); Intrinsic motivation is derived from the inner joy and satisfaction derived from a job (e.g. societal recognition and respect; appreciation shown by clients etc.).

Pooled baseline and follow-up staff responses showed that the predominant sources of motivation for staff in intervention facilities were intrinsic incentives including cordiality with clients and co-workers (mean = 3.5), career prospects (mean = 3.2) and staff strength/perceived workload (mean = 3.2). Staff in intervention facilities rated these motivation proxies higher than staff in control facilities (p = 0.000). Financial/extrinsic incentives were the least sources of motivation to staff particularly among staff in intervention facilities (see Fig 2), albeit the average monthly salaries/work allowances increased in nominal terms between 2012 and 2014, inflation not factored in.

Difference-in-difference estimations (see Table 2) confirmed the differences in sources of staff motivation in control and intervention facilities, after controlling the potential effect of staff age, gender, professional category, education, monthly salary, marital status, religion and health facility ownership. Moreover, the model specification for propensity score matching showed that staff in intervention facilities expressed relatively higher motivation levels than their counterparts in control facilities, especially in terms of intrinsic work incentives, career prospects, and perceived workload (p = 0.000) (see Table 3).

**Table 2:** Differences in staff motivation levels in treatment and control facilities.

		Baseline (2012)			Follow-up (2014)			Diff-in-Diff
		Treated	Control	Diff(BL)	Treated	Control	Diff(FU)	
Staff motivation factors^+^	^c^Obs	Mean(SE)	Mean(SE)	Mean(SE)	Mean(SE)	Mean(SE)	Mean(SE)	Mean(SE)
Physical work environment^++^	437	2.32(0.23)	2.33(0.21)	-0.01(0.01)	2.34(0.23)	2.25(0.22)	0.10(0.08)	0.11(0.12)
Financial and extrinsic incentives^a^	433	0.91(0.25)	1.10(0.23)	-0.19(0.01)*	1.14(0.23)	1.23(0.23)	-0.09(0.08)	-0.28(0.12)
Intrinsic incentives^b^	438	3.54(0.16)	3.32(0.16)	0.22(0.04) ‡	3.41(0.15)	2.97(0.16)	0.44(0.07) ‡	0.22(0.08)
Career prospects	431	2.84(0.23)	2.17(0.24)	0.68(0.11) ‡	2.87(0.25)	2.08(0.21)	0.79(0.10) ‡	0.11(0.17) ‡
Perceived workload	435	2.69(0.21)	2.19(0.22)	0.51(0.11) ‡	2.71(0.19)	2.20(0.20)	0.51(0.08) ‡	0.00(0.14) ‡
Overall motivation score	414	2.42(0.16)	2.17(0.15)	0.24(0.04) ‡	2.44(0.15)	2.11(015)	0.32(0.06) ‡	0.08(0.07) ‡

**Source:** WOTRO-COHEiSION Ghana Project (2014); Diff.-in-diff estimates*p<0.1; **p<0.05; ^‡^p<0.0001 (Means and SE are bootstrapped and estimated by linear regression).

^a^Extrinsic motivation is derived from financial and material work conditions of a job (e.g. salary increment, promotion, accommodation etc.).

^b^Intrinsic motivation is derived from the inner joy and satisfaction derived from a job (e.g. societal recognition and respect; appreciation shown by clients etc.).

^c^Number of pooled responses from baseline and follow-up surveys.

**Legend:** NOTE SE = Standard Error; All mean and SE values rounded up to the nearest decimal. FU = Follow-up survey; BL = Baseline survey.

^+^Motivation factors have been factor-analyzed.

^**++**^Physical work environment includes resource availability for staff to work with.

**Table 3:** Model specification for propensity score matching.

Variables	Variable definition	Intervention N = 103 (44%)		Control N = 131 (56%)		Difference in means
		Mean	SD	Mean	SD	
**Treatment variable**						
SCEI/NCEI	1 if SCEI clinic; 0 otherwise					
**Outcome variables**^+^						
Motivation factor 1	Motivation factors (factor-analyzed)	2.97	0.66	2.87	0.72	0.10
Motivation factor 2		1.80	0.64	1.90	0.70	-0.10
Motivation factor 3		3.63	0.32	3.29	0.53	0.34***
Motivation factor 4		3.19	0.51	2.51	0.91	0.68***
Motivation factor 5		3.24	0.49	2.71	0.72	0.52***
Overall motivation		2.96	0.33	2.66	0.46	0.30***
**Independent variables**						
Age	Staff age in years	37.4	13.1	37.6	13.1	-0.13
Gender	1 if male; 0 otherwise	0.40	0.49	0.30	0.46	0.10**
Education	1 if secondary education; 0 otherwise	0.28	0.45	0.21	0.40	0.07*
Profession	1 if non-clinical staff; 0 otherwise	0.26	0.44	0.24	0.44	0.02
Salary	1 if is >GHC 1,300; 0 otherwise	0.07	0.26	0.10	0.30	-0.03
Marital status	1 if married; 0 otherwise	0.44	0.50	0.45	0.50	-0.01
Religion	1 if Christian; 0 otherwise	0.97	0.18	0.96	0.20	0.01
Facility ownership	1 if private clinic; 0 otherwise	0.59	0.49	0.47	0.50	0.12**

**Source:** WOTRO-COHEiSION Ghana Project (2014); Note: SCEI = Systematic community engagement intervention; **Legend:** NSCEI = No Community engagement intervention; SD (standard deviation).

*p<0.1.

**p<0.05.

***p<0.001.

^+^Staff motivation factors (defined in methods section).

Results of propensity score matching established that the SCE interventions associated more with staff intrinsic motivation levels than financial incentives in both Greater Accra (ATT = 3.59, Pseudo R^2^ = 0.0401) and Western regions (ATT = 3.69, Pseudo R^2^ = 0.0765). Effect of the SCE interventions on financial incentives and physical work environment of staff was relatively low (see Table 4).

**Table 4:** Effect of community engagement interventions on staff motivation levels (n = 234).

Matching algorithm: Nearest Neighbor (NN)	Outcome indicators	^++^ATT (T-stat)	SE	Number of Intervention	Number of Control
Greater Accra region	Motivation factor 1	3.09(1.23)**	0.121	107	121
	Motivation factor 2	1.71(-1.27)**	0.136	104	120
	Motivation factor 3	3.59(3.57)**	0.070	104	120
	Motivation factor 4	3.05(3.74)**	0.154	107	120
	Motivation factor 5	3.25(4.89)**	0.115	107	119
	**Overall motivation**	2.93(3.37)**	**0.076**	**116**	**104**
Western region	Motivation factor 1	2.88(0.39)*	0.148	101	114
	Motivation factor 2	1.92(-0.94)*	0.130	99	116
	Motivation factor 3	3.69(2.89)*	0.095	101	117
	Motivation factor 4	3.35(6.42)*	0.167	99	111
	Motivation factor 5	3.23(3.05)*	0.129	100	115
	**Overall motivation**	3.01(2.85)*	**0.088**	**96**	**104**

**Source:** WOTRO-COHEiSION Ghana Project (2014).

* Pseudo R^2^<1.0.

** Pseudo R^2^ <0.05.

^**++**^ATT (Average treatment effect on the treated). The ATT values are the propensity score matching output and they depict the impact of the treatment (SCE interventions) on each of the staff motivation markers, high values imply higher treatment effect and *vice versa*.

**Legend:** SE (Standard Error); Motivation factor 1 (Physical work environment and resource availability); Motivation factor 2 (Financial/extrinsic incentives); Motivation factor 3 (Intrinsic incentives including cordiality with clients and co-workers); Motivation factor 4 (Career prospects); Motivation factor 5 (Perceived workload and staff availability); Overall motivation (Overall score based on all five motivation factors).

Overall, the findings suggest SCE activities enhanced mutual collaboration and relationships between healthcare staff and clients but had minimal effect on financial/extrinsic motivation because the study did not have the capacity to influence increment of staff salaries and other financial incentives.

### Association between community groups and staff motivation factors

Sub-sample analysis of only follow-up data of intervention facilities showed that staff motivation levels appeared to have some association with the different community groups involved in the SCE interventions. As shown in Table 5, healthcare facilities assessed by community artisan groups (Coef. = 0.2268, p = 0.0368) and female groups (Coef. = 0.2720, p = 0.0118) appeared to have more intrinsically motivated staff and perceived better the cordial relationship between clients and staff.

**Table 5:** Association between community groups and staff motivation factors at follow-up (n = 103).

Groups characteristics		Staff motivation factors (factor-analyzed)					
		Factor 1	Factor 2	Factor 3	Factor 4	Factor 5	Overall
Group type	N	Coef.	Coef.	Coef.	Coef.	Coef.	Coef.
Religious	22	0.0200	-0.2333**	-0.0528	-0.3476**	0.0996	-0.1557
Traders	8	0.0508	0.2165**	-0.0531	0.1678	-0.0371	0.0789
Widows	1	0.0724	0.0232	0.0596	0.0899	-0.1672	0.0526
Community volunteers	3	0.0859	0.1799	0.1322	0.1422	0.0264	0.1724
Music	2	0.1689	-0.0458	0.0788	-0.0256	0.0266	0.0494
Artisans	5	0.1578	-0.0298	0.2268*	-0.0935	-0.0245	0.1481
Youth	11	-0.3488**	0.0019	-0.1681	0.2281**	-0.0621	-0.1293
**Gender distribution**							
All males	2	-0.0140	-0.0627	-0.0489	-0.0518	0.0066	-0.0579
All females	5	0.1478	0.0319	0.2720**	0.1381	-0.0915	0.2557**
Male dominant	13	-0.1051	0.1802	0.0498	0.0965	0.1043	0.0622
Female dominant	31	0.0637	-0.1286	-0.1782	-0.1604	-0.0137	-0.1290
Equal males and females	1	-0.1624	-0.1230	0.0596	0.0899	-0.1672	-0.1650
**Age distribution**							
Youthful (18–30 years)	18	-0.0039	0.1205	0.0411	0.1949*	0.1384	0.1812
Elderly (31+ years)	34	0.0039	-0.1205	-0.0411	-0.1949*	-0.1384	-0.1812
**Education**							
Mainly illiterates/uneducated	11	0.1816*	0.1190	0.1499	0.1830*	0.0420	0.2570**
Mainly literates/educated	12	0.0181	0.0283	-0.0081	0.2092	0.0362	0.0240
Literates and illiterates	29	-0.1547	-0.1151	-0.1041	-0.3318**	-0.0649	-0.2168*
**Location**							
Rural	28	-0.0350	-0.0142	0.0691	0.0508	-0.1510	-0.0164
Urban	24	0.0350	0.0142	-0.0691	-0.0508	0.1510	0.0164
**Leadership/Organization**							
Structured	39	0.1002	0.1669	0.0701	0.1702	0.0117	0.1959*
*Ad hoc*	13	-0.1002	-0.1669	-0.0701	-0.1702	-0.0117	-0.1959*
**Meeting dynamics**							
Group size (mean = 29)	52	0.0495	0.1020	0.1481	-0.0588	-0.2268**	0.0261
Attendance rate (mean = 60%)	52	-0.1478	-0.0434	-0.0211	0.0317	-0.0651	-0.1571
^a^Meeting duration (mean = 41)	52	-0.1561	-0.0135	0.0599	0.0873	-0.2808*	-0.1156
^b^Time per participant (mean = 1.4)	52	-0.1087	-0.0753	-0.1250	0.0720	0.0603	-0.1107

**Source:** WOTRO-COHEiSION Ghana Project (2014); Spearman rank correlation test *p<1.0; **p<0.05

**Legend:** Motivation factor 1 (Physical work environment and resource availability); Motivation factor 2 (Financial/extrinsic incentives); Motivation factor 3 (Intrinsic incentives including cordiality with clients and co-workers); Motivation factor 4 (Career prospects); Motivation factor 5 (Perceived workload and staff availability); Overall motivation (Overall score based on all five motivation factors).

Healthcare facilities assessed by traders groups seemed to favor higher extrinsic/financial motivation ratings by staff (Coef. = 0.2165, p = 0.0494). Staff motivation by physical work environment had a negative association with youth groups (Coef. = -0.3488, p = 0.0010) but positively associated with literate/educated community groups (Coef. = 0.1816, p = 0.0942). Community group size (Coef. = -0.2268, p = 0.0357) and meeting duration (Coef. = -0.2808, p = 0.0088) appeared to associate negatively with staff perception ratings on workload/staff availability (see Table 5). These revelations could be explored by future researchers to ascertain possible reasons for the associations.

## Discussion

Community engagement in health is not new to Ghana’s healthcare system [35–39] though the concept is often not applied in the context of health worker motivation. In Ghana, staff motivation policies often emphasize extrinsic incentives such as salary increment, payment of extra duty hour allowance, rural/deprived area allowance, early promotion and provision of staff accommodation to improve staff motivation levels [40]. Even though these interventions are relevant, their impact on staff motivation levels to stimulate retention, productivity and quality service delivery remain debatable [5,7]. Moreover, the cost implications of sustaining public sector wage increases compels the need to explore potential benefits of promoting non-financial incentives and mutual collaboration between healthcare providers and clients through community engagement.

As demonstrated in this study, community engagement in healthcare quality assessment could enhance client-provider relationships and potentially improve intrinsic motivation levels of staff. Findings in this study resonate with conclusion by Källander et al [41] that participatory community engagement in health programmes has a positive association with staff motivation and retention outcomes. Källander et al [41] arrived at this conclusion following a randomized control trial conducted in Uganda and Mozambique on the effect of innovative staff motivation and supervision approaches on community health worker performance and retention.

Similar studies in Ghana [36,42] and elsewhere [16, 20,43,44] have alluded to the increasing relevance of mutual collaboration and engagement between communities and health providers. These empirical evidences reinforce the argument that non-financial incentives play a critical role in health worker motivation.

A study by Dil et al [42] on motivation and challenges of community-based surveillance volunteers in northern Ghana found that the community was as a vital motivating factor for staff in terms of altruism, sense of duty to the community, gaining community respect and pride. Dil et al [42] found that payment of financial rewards were not emphasized though recognized as vital to help attain basic needs. Similar studies on Ghana [5,7,45] indicated that even though health staff recognized monthly salaries as important work incentives, they often attributed their motivation and retention decisions to the desire to help the community.

As illustrated in this study, promoting community engagement in health has the potential to enhance staff experiences and work relationship with clients while encouraging better health seeking behavior by clients. For instance, it was found that staff in intervention facilities experienced relatively lower cases of self-medication by clients than staff in control facilities (see Fig 1). Moreover, the average number of clients defaulting in treatment protocols per month was lower in intervention health facilities. Bhutta et al [46] made similar observations following a community-based study in Pakistan; it was found that stillbirths and neonatal mortality rates reduced in two sub-districts where community-based strategies between healthcare providers and community members were implemented.

An important counter intuitive observation in this study was the high number of medical complications due to client late reporting and number of clients seeking care from informal caregivers (i.e. faith healers, spiritualists and traditional/alternate medicine practitioners). Although frequency of community outreaches per month had increased in both control and intervention facilities, the increases did not seem to translate into better health seeking behaviours of clients (see Fig 1). Perhaps these community outreaches have not been effective in addressing barriers to timely health service utilization.

Furthermore, challenges confronting the NHIS in recent times relating to delayed provider reimbursements [47,48] might explain the poor client health seeking behavior. The delayed provider reimbursements have reportedly compelled some accredited healthcare providers to resort to co-payment especially for drugs and medical laboratory services [49]. This emerging phenomenon potentially reduced financial accessibility to formal healthcare hence reliance on informal caregivers for health services.

Informal caregivers appeared to be the first port of call for many community members who are only compelled to visit orthodox healthcare providers when their condition worsens. This poor health seeking behavior of clients is partly attributed to poor staff attitudes and human relations with clients often induced by low motivation levels and morale at work [12,48–51]. Similar studies in Mozambique suggest that health worker motivation and their relationship with community members influence clients’ service utilization and access to service quality. Audet et al [52] found that poor-quality health services and lack of programme support resulted in low uptake of HIV testing in rural Mozambique.

These findings denote that besides instituting stringent disciplinary actions against staff indulged in unprofessional practices it will be beneficial to promote mutual collaboration between clients and health providers through community engagement strategies.

Unfavorable work conditions, including non-financial incentives are also cited as key contributory factors for poor staff attitudes toward clients and unwillingness to work in rural deprived areas. A study involving 3,199 medical and nursing students in Asia and Africa found that 28% (870/3156) of the respondents intended to migrate abroad, and only 18% (575/3158) intended a rural career after training [53].

As part of efforts to reverse this trend, more stringent staffing norms should be enforced to ensure that the right caliber of health trainees willing to accept postings to rural areas are recruited for training. Moreover, the WHO global code of practice on the International Recruitment of Health Personnel (2015) encourages under-staffed health systems to train and retain the health personnel they need to limit demand for international migration [54].

As shown in this study, staff monthly salaries and other financial incentives seemed to have improved overtime in nominal terms but this did not appear to influence positive client health seeking behavior neither did it serve as a key source of motivation for staff (see Table 2). This observation contrasts anecdotal and some empirical [10,12] notions that financial incentives are the fundamental determinants of staff motivation and retention in Ghana.

Authors of this paper do not seek to advocate against financial incentives because they remain critical for attracting and retaining qualified health professionals to render quality healthcare services to clients [6,10,12]. The argument is that mainly depending on financial incentives as a strategy for motivating healthcare workers might be a challenging trajectory for Ghana to sustain in the midst of already constrained health budgets.

Considering the global differentials in financial incentives for health workers it is not likely Ghana can compete with the global health labour market to motivate and retain health workers [55] through wage increases alone hence the need to adopt a balanced approach. Albeit public sector wage bill accounts for over 90% of government domestic expenditure on health [47], staff productivity and quality of healthcare delivery remain a challenge [3,5,6,7].

In view of the above findings, there is the need to identify and execute non-financial work incentives to complement existing efforts towards enhancing staff motivation. In the past, attempts by the ministry of health in Ghana to use rural financial incentives to promote staff motivation did not yield the needed results and had to be curtailed for repackaging [55].

As demonstrated in this paper, non-financial work incentives such as cordiality with clients and co-workers formed an important basis for staff motivation in primary healthcare facilities.

Design and implementation of staff motivation packages should recognize that “wholesale” work incentives mainly based on financial rewards might not ultimately results in intrinsically motivated staff. Instead, staff motivation packages should be tailor-made to the peculiar needs of staff at various levels of the healthcare system.

Findings in this study should stimulate policy dialogues among relevant stakeholders of Ghana’s healthcare system on the prospects of using community engagement [56] to enhance staff intrinsic motivation levels and experiences with clients.

It is recommended that staff appraisal protocols for the public and private health sectors in Ghana should be discussed for possible reforms to enable community members provide feedback on staff performance and relationship with clients. Community engagement sessions at the community level could serve as the platform for these feedbacks and possibly count towards staff annual performance appraisal and subsequent promotion. This idea will help promote accountability and good working relationship with clients.

Existing community-based programmes such as the Community-based Health Planning and Services (CHPS) programme could constitute the framework for a pilot of the proposed community-based staff appraisal system. This proposal could be piloted on a small scale after wider stakeholder consultation to ascertain its feasibility and sustainability. Anonymity of the clients should be assured during appraisal to avoid potential intimidation.

As demonstrated in this study, the types of community groups engaged in the SCE interventions had associations with the staff motivation ratings. High intrinsic motivation levels were particularly associated with community artisan groups, female groups and groups with structured leadership. This positive association corroborates the assertion that females in Ghana turn to have more health needs and demand for healthcare services than males [56]. This implies females could be better assessors of healthcare quality because their encounter with healthcare staff is more frequent than their male counterparts.

Community group dynamics should be adequately explored when implementing the SCE concept to guarantee effectiveness and sustainability. The findings also suggest informal community groups with organized leadership are better options for effective SCE activities. Healthcare providers will most likely implement quality improvement recommendations from organized community groups than groups engaged in *ad hoc* activities without clear leadership structure.

### Limitations

The study was conducted in clinics and health centres where relatively lower cadres of health staff work. It is possible staff experiences and motivation ratings reported in this paper were largely influenced by the focus on these staff. Work conditions are usually poorer in lower level health facilities than secondary and tertiary level facilities.

Furthermore, institutional and national level developments (beyond the control of this study) might have occurred between the baseline and follow-up period and possibly affect health staff experiences/motivation levels in healthcare facilities. Health facilities that were upgraded to higher level facilities during follow-up might have recorded higher numbers of clinic attendance which has the potential to influence motivation level and staff experiences in terms of workload and relationship with clients.

Moreover, interviewed health staff were not randomized into the intervention and control facilities hence predisposing the study to selection bias [30]. Cognizant of this potential bias, propensity score matching and difference-in-difference estimations were used to control the possible effect of covariates on staff responses and determine effect of the interventions. Overall, the authors acknowledge that this study would have been much stronger as a multiple methods research but limited financial resources and time did not permit extensive application of all relevant research methodologies.

## Conclusion

Community-based approach to health worker motivation is a potential complementary strategy that needs policy deliberation to explore its prospects. Even though financial incentives remain critical sources of motivation that should not be compromised, mainly depending on these incentives might not promote intrinsic motivation levels among staff as demonstrated in this study. Health workers’ commitment to quality healthcare delivery will most likely be enhanced when they are motivated intrinsically through mutual collaboration with clients.

Also, effective collaboration between healthcare providers and communities is needed to promote client trust and confidence in the formal healthcare system in Ghana and ultimately improve universal access to basic healthcare services. The critical role of female groups in promoting staff-client cordiality and relationships has been established in this study and should be explored in future studies and policy deliberations on health worker motivation strategies.

## Abbreviations

ERC: Ethical Review Committee
CHPS: Community-based Health Planning and Services
CHVs: Community Health Volunteers
GAR: Greater Accra Region
GHS: Ghana Health Services
LMICs: Low Income Countries
MoH: Ministry of Health
NHIA: National Health Insurance Authority
NHIS: National Health Insurance Scheme
OPD: Out-patient Department
PCA: Principal Component Analysis
PBF: Performance-based Financing
WHO: World Health Organization
WR: Western Region
SCE: Systematic Community Engagement
